# High-Throughput Characterization of Viral and Cellular Protein Expression Patterns During JC Polyomavirus Infection

**DOI:** 10.3389/fmicb.2019.00783

**Published:** 2019-04-17

**Authors:** Jeanne K. DuShane, Michael P. Wilczek, Mason A. Crocker, Melissa S. Maginnis

**Affiliations:** ^1^Department of Molecular and Biomedical Sciences, The University of Maine, Orono, ME, United States; ^2^Graduate School in Biomedical Sciences and Engineering, The University of Maine, Orono, ME, United States

**Keywords:** JC polyomavirus, high-throughput, infectivity, protein expression, near-infrared detection

## Abstract

JC polyomavirus (JCPyV) is a ubiquitous human pathogen and the causative agent of a fatal demyelinating disease in severely immunocompromised individuals. Due to the lack of successful pharmacological interventions, the study of JCPyV infection strategies in a rapid and highly sensitive manner is critical for the characterization of potential antiviral therapeutics. Conventional methodologies for studying viral infectivity often utilize the detection of viral proteins through immunofluorescence microscopy-based techniques. While these methodologies are well established in the field, they require significant time investments and lack a high-throughput modality. Scanning imager-based detection methods like the In-cell Western (ICW)^TM^ have been previously utilized to overcome these challenges incurred by traditional microscopy-based infectivity assays. This automated technique provides not only rapid detection of viral infection status, but can also be optimized to detect changes in host-cell protein expression during JCPyV challenge. Compared to traditional manual determinations of infectivity through microscopy-based techniques, the ICW provides an expeditious and robust determination of JCPyV infection. The optimization of the ICW for the detection of viral and cellular proteins during JCPyV infection provides significant time and cost savings by diminishing sample preparation time and increasing resource utilization. While the ICW cannot provide single-cell analysis information and is limited in the detection of quantitation of low-expressing proteins, this assay provides a high-throughput system to study JCPyV, previously unavailable to the field. Thus, the high-throughput nature and dynamic experimental range of the ICW can be applied to the study of JCPyV infection.

## Introduction

JC polyomavirus (JCPyV), the etiological agent of the fatal demyelinating disease progressive multifocal leukoencephalopathy (PML), infects between 50–80% of the human population (Egli et al., 2009; Kean et al., 2009). Spread via peroral transmission, JCPyV establishes primary infections in the host, specifically in the kidneys and B lymphocytes, producing an asymptomatic infection in healthy individuals (Dubois et al., 1997; Dorries et al., 2003; Chapagain and Nerurkar, 2010). However, under conditions of severe immunosuppression, JCPyV can spread from primary sites of infection into the central nervous system (CNS), targeting both astrocytes and oligodendrocytes for infection (Silverman and Rubinstein, 1965; Zurhein and Chou, 1965; Kondo et al., 2014). The lytic infection of these glial cell types leads to CNS demyelination and the onset of PML, for which there are currently no effective therapies (Ferenczy et al., 2012).

JC polyomavirus is a member of the *Polyomaviridae* family of viruses, which also includes simian virus 40 (SV40), one of the most widely studied viruses (DeCaprio and Garcea, 2013). Polyomaviruses have a double-stranded DNA genome enclosed within a proteinaceous capsid comprised of viral proteins 1 (VP1), 2, and 3 (Ferenczy et al., 2012). VP1 serves as the viral attachment protein that initiates binding to the JCPyV receptor α2,6-linked lactoseries tetrasaccharide c (LSTc) (Neu et al., 2010; Maginnis et al., 2013; Stroh et al., 2015). Viral binding alone is not sufficient to support infection, as viral internalization requires the 5-hydroxytryptamine 2 subfamily of receptors (5-HT_2_Rs) (Elphick et al., 2004; Assetta et al., 2013). Internalization is mediated by clathrin-dependent endocytic events, trafficking through the endocytic compartment to the endoplasmic reticulum, and finally deposition into the nucleus (Querbes et al., 2004, 2006; Nelson et al., 2012; Assetta et al., 2013). While the initial stages of SV40 infection vary slightly from JCPyV, all polyomaviruses traffic into the ER prior to genomic deposition in the nucleus. Early viral genes, such as the T-antigens, are transcribed first followed by DNA replication and the transcription of viral late genes that encode the capsid proteins. New viral progeny are then encapsidated and eventually egress from the host cell (Loeber and Dorries, 1988; Ault and Stoner, 1993; Agostini et al., 1997; Bhattacharjee and Chattaraj, 2017).

While significant advances have been made in the characterization of JCPyV replication strategies, JCPyV research productivity has been hindered by the lack of a productive animal model and limited cellular tropism (Zu Rhein and Varakis, 1979; Houff et al., 1983). Currently, the study of JCPyV relies heavily on manual microscopic analysis of cell-culture-based assays to characterize infectivity (Assetta and Atwood, 2017). The most widely used assay to measure JCPyV infectivity is the fluorescent focus unit (FFU) assay, which requires viral protein-specific antibodies to label infected cells for detection via epifluorescence microscopy (Payne et al., 2006; Calgua et al., 2011). While the FFU assay is a reliable and well-characterized virological method, it presents several challenges: it can introduce observer bias, often relies on partial sample analysis to generate representative data, and requires a significant time investment that reduces research productivity and limits the feasibility of large-scale screens. To address these issues, other technologies, like the pseudovirus system, have been generated to enable high-throughput data collection of JCPyV infectivity (Pastrana et al., 2004; Gee et al., 2013). However, this system relies on virus-like particles that lack infectious DNA, and thus can only provide insights into the early steps in the viral lifecycle. In contrast, the In-Cell Western (ICW)^TM^ assay, has been shown to effectively quantitate viral infection using infectious viruses like influenza, herpes simplex virus, reovirus, and rotaviruses by employing a high-throughput laser-based scanning technology (Wan et al., 2010; Iskarpatyoti et al., 2012; Fabiani et al., 2017). The ICW employs a similar method to that of indirect immunofluorescence staining, yet utilizes a secondary near infrared (NIR)-conjugated antibody labeling system. Data regarding viral infection can then be obtained with this automated infrared imaging system, eliminating observational biases and greatly reducing the time needed to reliably quantitate infectivity data.

The goal of this study was to adapt the ICW assay as a reliable method to enable high-throughput study of JCPyV infection to enhance the rate of discovery and improve the feasibility of large-scale screens. To this end, the ICW has been shown to accurately characterize JCPyV infectivity at variable levels of infection, including viral inhibition through chemical and siRNA treatments, and for the quantification of host-cell protein expression during viral challenge. These findings demonstrate that the ICW assay provides an effective measure of viral infection and can be utilized as a platform for high-throughput screening of JCPyV infectivity.

## Materials and Methods

### Cell Types and Viruses

Human fetal glial SVG-A cells were cultured in Minimum Essential Medium (Corning) supplemented with 10% fetal bovine serum (FBS) (Atlanta Biologicals), 1% penicillin/streptomycin (P/S) (Mediatech, Inc.), and 0.2% Plasmocin prophylactic (Invivogen) (cMEM). Cells were maintained in a humidified incubator at 37°C with 5% CO_2_. SVG-A cells (Major et al., 1985) were generously provided by the Atwood laboratory (Brown University) and have been authenticated by ATCC through STR profiling. Viral infections were performed with a purified JCPyV strain Mad-1/SVEΔ as previously described (Nelson et al., 2012), or SV40 strain 777. Virus strains were generously provided by the Atwood laboratory.

### Cell Stains and Antibodies

Commercially available ab34756 (Abcam) and a monoclonal antibody derived from a hybridoma supernatant (PAB597) (generously provided by the Atwood laboratory) (Maginnis et al., 2013) were used to probe for the JCPyV major capsid protein VP1 to quantitate JCPyV infection. PAB597 cross-reacts with the SV40 VP1 protein (Maginnis et al., 2013) and was also used to score SV40 infectivity. Antibodies for ERK1/2 (ERK) (CST #4695), phosphorylated ERK1/2 (pERK) (CST #9101), or GAPDH (Abcam, ab8245) were used for ICW or western blot protein quantifications. For the ICW, LI-COR 800 anti-mouse or anti-rabbit secondary antibodies (LI-COR) were used. For FFU, Alexa Fluor 488 (Thermo Fisher Scientific) anti-mouse or anti-rabbit secondary antibodies were used. CellTag 700 (LI-COR) was used as a cell count normalization stain for ICW assays as indicated. DAPI nuclear counterstain (Thermo Fisher Scientific) was used as a cell count normalization stain for FFU assays.

### Chemical Treatments and siRNAs

Chemical inhibitors of MEK, U0126 and PD98059 (Cell Signaling Technology), were used at concentrations of 10 and 50 μM, respectively. Bay43-9006 (Cayman Chemical), a chemical inhibitor of Raf, was used at 15 μM. Retro2 (Sigma–Aldrich), a retrograde trafficking inhibitor, was used at a concentration of 100 μM (Nelson et al., 2013). All chemical inhibitors were reconstituted in DMSO (Cell Signaling Technology), which served as a volume-specific vehicle control. EGFR and ERK1/2 siRNAs (Cell Signaling Technology) were transfected into SVG-A cells with RNAiMax (Thermo Fisher) at 10 pmol per well per manufacturer’s instructions. Successful transfections of the siRNAs were confirmed using BLOCK-iT Red (Thermo Fisher) (DuShane et al., 2018).

### JCPyV and SV40 Infectivity at Varying MOIs

SVG-A cells were infected with JCPyV and SV40 at MOIs indicated per figure legend in cMEM at 37°C for 1 h. Cells were then fed with cMEM, and plates were incubated at 37°C for 72 h. At 72 hpi, cells were washed with 1X PBS, fixed in 4% PFA at RT for 10 min, and washed three times in 1X PBS prior to staining for either ICW or FFU.

### Chemical Inhibition of JCPyV Infectivity

SVG-A cells were plated to ∼70% confluency in 96-well plates. Cells were pre-treated with cMEM containing DMSO, U0126, PD98059, Bay43-9006, or Retro2 at 37°C for 1 h. Following pre-treatment, cells were infected with JCPyV (MOI = 0.5 FFU/cell) at 37°C for 1 h. Media containing the indicated treatment was then added back to appropriate wells at 37°C for 72 h with the exception of Bay43-9006-treated wells. Bay43-9006-treated wells were incubated for at 37°C for 2 h following infection, aspirated, washed with 1X PBS, and cMEM was added back for the duration of the experiment. At 72 hpi, cells were fixed in 4% PFA, washed with 1X PBS three times and FFU and ICW plates were stained as described.

### siRNA Inhibition of JCPyV Infectivity

Prior to siRNA transfection, SVG-A cells were plated to ∼50% confluency in 12-well plates (Greiner Bio-One). Cells were then transfected with an EGFR or ERK1/2 siRNA with RNAiMax. The RNAiMax transfection reagent and siRNAs were diluted in incomplete MEM (lacking FBS and antibiotics), combined, and incubated at RT for 5 min. siRNA complexes were added to SVG-A cells and incubated at 37°C for 72 h. At 72 hpt, cells were infected with JCPyV (MOI = 0.5 FFU/cell) at 37°C for 1 h. Cells were then fed with cMEM and incubated at 37°C for 72 h. At 72 hpi, cells were fixed with 4% PFA, washed with 1X PBS three times, and stained for VP1 for FFU and ICW assays as described.

### Fluorescent Focus Unit Assay Staining and Quantitation of Viral Infection

Following fixation, cells were permeabilized with 1X TBS-1% Triton X-100 at RT for 15 min and were then incubated with TBS Odyssey Blocking Buffer (LI-COR) at RT for 1.5 h. Cells were stained with the primary antibodies PAB597 (JCPyV or SV40 VP1, 1:40) or ab34756 (JCPyV VP1, 1:1000) in TBS Odyssey Blocking Buffer (LI-COR) at 4°C overnight while rocking. After primary incubation, cells were washed with 1X PBS and incubated with either an anti-mouse or anti-rabbit Alexa Fluor polyclonal 488 antibody (Thermo Fisher Scientific) at RT for 1 h while rocking and nuclei were counter stained with DAPI (Thermo Fisher Scientific). Using a Nikon Eclipse Ti epifluorescence microscope (Micro Video Instruments, Inc.), the number of infected cells per 10x visual field was quantitated. Percent infection was determined by dividing the number of infected cells/field by the total number of DAPI-positive nuclei/field as previously described (DuShane et al., 2018), reported as percent infection. As indicated, the average percent infection was normalized to the highest MOI, vehicle control DMSO, or siRNA controls (set at 100%).

### ICW Verification of Host-Cell Protein Knockdown

To confirm ERK1/2 host-cell protein knockdown by ICW, SVG-A cells transfected with EGFR and ERK1/2 siRNAs (as described) were fixed at 72 hpt with 4% PFA. Cells were washed with 1X PBS three times and stained for total ERK and CellTag for ICW analysis with the LI-COR Odyssey CLx.

### In-Cell Western Assay Staining and Protein Quantification

Following fixation, cells were incubated with 1X TBS-1% Triton X-100 to permeabilize for 15 min. Cells were then incubated with TBS Odyssey Blocking Buffer (LI-COR) at RT for 1.5 h while rocking. Cells were stained with primary antibodies as indicated: PAB597 (1:40), ab34756 (1:1000), ERK1/2 (1:500), or pERK1/2 (1:500) in TBS Odyssey Blocking Buffer (LI-COR) at 4°C overnight while rocking. After primary incubation, cells were washed with 1X TBS-T and incubated with either an anti-mouse or anti-rabbit LI-COR 800 secondary antibody (1:10,000) and CellTag 700 (1:500) at RT for 1 h while rocking. Secondary-alone wells were treated only with species-appropriate LI-COR 800 secondary antibody (1:10,000). Cells were washed with 1X TBS-T three times and aspirated to remove all liquid prior to scanning. Using a LI-COR Odyssey CLx Infrared Imaging system, plates were immediately scanned to detect 700 and 800 nm channel intensities. Plates were read at a 42 μm resolution, at medium quality, with a 3.0 mm focus offset. After scanning, 700 and 800 nm channels were aligned using the Image Studio software (version 5.2) equipped with the ICW module. After scanning, the ICW analysis grid (Image Studio) was applied to the plate image to outline each well and images were then processed using Image J (NIH).

### Western Blot Verification of Host-Cell Protein Knockdown

Western blot analysis of ERK1/2 protein expression was also used to confirm siRNA protein knockdown. SVG-A cells were transfected with either EGFR or ERK1/2 siRNAs as described above. Cells were then washed with 1X PBS and manually scraped from sample wells. Cells were pelleted by centrifugation at 376 ×*g* 4°C for 5 min. Pellets were resuspended in 50 μl of Tris-HCl lysis buffer containing protease and phosphatase inhibitors and incubated on ice for 15 min. Samples were centrifuged at 18,600 ×*g* at 4°C for 10 min. Samples were combined with Laemmli sample buffer (Bio-Rad), boiled at 95°C for 5 min, and proteins were then resolved by SDS-PAGE using a 4–15% gel (Bio-Rad). Proteins were transferred onto a nitrocellulose membrane with a Transblot Turbo Transfer System (Bio-Rad). Protein-containing membranes were then blocked with 5% non-fat dry milk/TBS-T (1X TBS/0.1% Tween 20) overnight at 4°C while rocking. Membranes were washed with 1X TBS-T for 15 min, three times. Membranes were then incubated with primary antibodies for total ERK (1:500) and GAPDH (1:2000) in 5% BSA/TBS-T overnight at 4°C while rocking. After primary antibody incubation, membranes were washed in TBS-T at RT for 15 min three times each and then incubated with the secondary anti-rabbit 680 antibody (1:10,000) (LI-COR) and anti-mouse 800 antibody (1:10,000) (LI-COR) at RT for 1 h in 5% milk/TBS-T. Membranes were washed in TBS and then imaged using a LI-COR Odyssey CLx.

### JCPyV-Induced Activation of ERK

SVG-A cells were plated to ∼70% confluency in 96-well plates. Cells were either mock-infected (cMEM only) or infected with JCPyV (MOI = 0.5 FFU/cell) in cMEM at 37°C for 0, 15, 30, or 60 min. At the indicated timepoints, cells were fixed in 4% PFA at RT for 10 min and washed three times in 1X PBS. After fixation, both mock- and JCPyV-infected wells were probed for pERK and CellTag for ICW analysis with the LI-COR Odyssey CLx.

### Image J Analysis of ICW-Plate Images

Each plate processed for ICW was scanned using the LI-COR Odyssey CLx imager, and a size-specific plate template was added to the image containing both 700- and 800-channel intensities to define well boundaries. Each image was subsequently loaded into ImageJ for analysis, adapted from previous models (Rueden et al., 2017; Kelley and Paschal, 2018). In brief, RGB ICW image channels were split and a background subtraction was applied to the 8-bit, red and green channels, in accordance to well size, using the rolling ball radius algorithm. To measure signal intensity within each well, a mask of each well was generated from the aforementioned size-specific template as regions of interest (ROIs). The ROIs generated from the template image were then applied to both the 700 (red) and 800 (green) images, and the fluorescence intensities from each well (as characterized per pixel) were generated. To account for background fluorescence intensity from uninfected or untreated wells (mock), the mock sample (as experimentally indicated) intensity values for both the 700 and 800 channels were subtracted from experimental wells to account for non-specific fluorescence. Resultant values were plotted using the ggplot2 R package (version 3.5.1) (Wickham, 2009) and reported as percent response.

### Statistical Analyses

Student’s t-test was used to compare means from at least triplicate samples in Microsoft Excel to determine statistical significance. P-values < 0.05 were considered statistically significant. Each experiment was performed in triplicate containing a minimum of triplicate samples. Pearson correlation coefficients, calculated using R, were used to correlate manual microscopy FFU (percent infection) data with corresponding ICW (percent response) data as indicated.

## Results

### Characterization of JCPyV Infectivity by ICW

**FIGURE 1:**
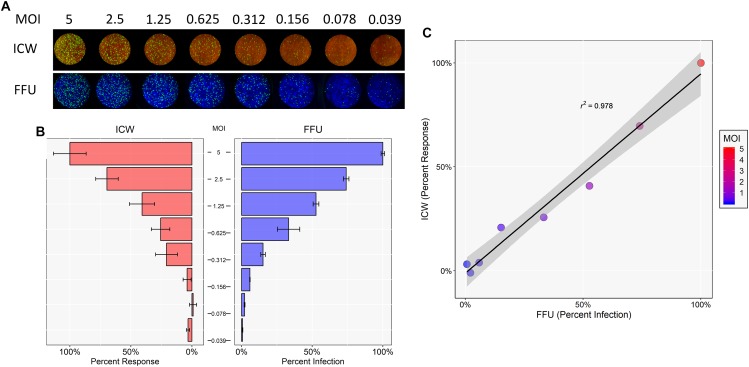
Quantitation of JCPyV infection by FFU and ICW. SVG-A cells were infected with JCPyV at indicated MOIs (FFU/cell) at 37°C for 1 h. At 1 hpi, cells were fed with cMEM and incubated for 72 h. At 72 hpi, cells were fixed and stained for VP1 expression. JCPyV VP1 expression was analyzed by ICW and FFU quantitation. **(A)** ICW plates were stained with CellTag 700 (red) and a LI-COR-800 secondary antibody to target VP1 (green). ICW infectivity (percent response) was quantified using a LI-COR Odyssey CLx infrared imager equipped with Image Studio software and processed with ImageJ. FFU plates were stained for VP1 and counterstained with DAPI as a cell count normalization control. **(B)** FFU quantification of VP1 (percent infection) was scored using a Nikon epifluorescence microscope and NIS Elements software. For each experimental assay, a minimum of triplicate samples were analyzed and the percent infection or percent response of a given MOI was normalized to the highest MOI, which was set to 100%. Error bars are representative of the standard deviation from the mean. **(C)** ICW and FFU data were compared across identical conditions by linear regression and an experimental correlation was determined using a Pearson correlation coefficient. The gray shaded region represents the confidence interval (standard error of the linear regression). Data are representative of three independent experiments.

To determine whether the ICW can be used to accurately quantitate JCPyV infection, SVG-A cells were infected with varying JCPyV MOIs and analyzed for infectivity using manual FFU quantitation (percent infection) and ICW (percent response) analyses performed in parallel (Figure 1A,B). The Cell Tag 700 (ICW) and DAPI stain (FFU) were used as total cell number normalization controls to quantify the percentage of infected cells. Infectivity was characterized through measurement of newly synthesized VP1 at 72 hpi, which represents a single replication cycle. Both the percent infection and percent response data demonstrate an increase in VP1 expression with increasing MOIs. Interestingly, the percent infectivity and percent response data were positively correlative across the range of the varying MOIs tested, as demonstrated by a Pearson correlation coefficient of 0.978 (Figure 1C). These data indicate that the ICW is a robust and accurate assay for scoring JCPyV infectivity.

### Quantification of JCPyV Infectivity by ICW Following Chemical Inhibition

A key protein necessary for productive JCPyV infection is the host-cell protein extracellular signal-regulated kinase (ERK), a member of the mitogen-activated protein kinase (MAPK) cascade. It has been previously shown that chemical inhibition of ERK significantly reduces JCPyV infection (DuShane et al., 2018). ERK functions as the terminal kinase of the MAPK cascade and is directly phosphorylated by MEK, which is activated by the kinase Raf (Shaul and Seger, 2007). It has been previously shown that inhibitors of MEK (U0126 and PD98059) can be applied to cultured cells to inhibit the activation of the downstream target ERK (English and Cobb, 2002; Wan et al., 2004), thereby impacting JCPyV infection (Ravichandran et al., 2007; DuShane et al., 2018). Moreover, as Raf is critical for the downstream activation of ERK, chemical inhibition of Raf (Bay43-9006) was also investigated to determine if viral infection was impacted.

**FIGURE 2:**
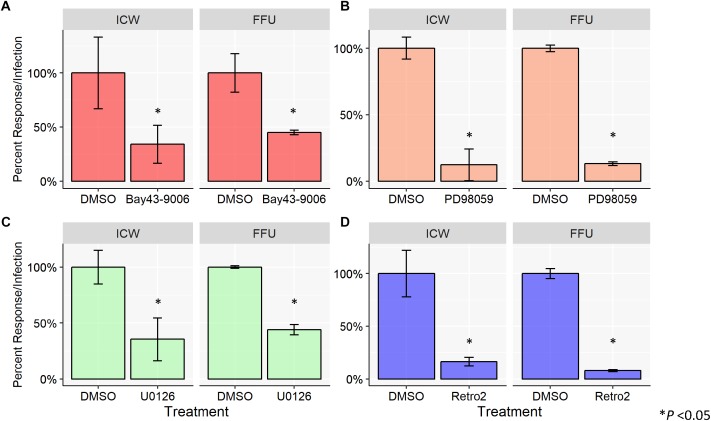
Chemical inhibition of host-cell protein activation inhibits JCPyV infection. SVG-A cells were pre-treated with cMEM containing either DMSO (volume specific vehicle control), **(A)** Bay43-9006 (15 μM), **(B)** PD98059 (50 μM), **(C)** U0126 (10 μM), or **(D)** Retro2 (100 μM) at 37°C for 1 h. Cells were then infected with JCPyV (MOI = 0.5 FFU/cell) at 37°C for 1 h. At 1 hpi, treated media was added back to the appropriate wells for 72 h with the exception of wells with Bay43-9006-containing medium, which was removed at 2 hpi, and replaced with cMEM for the duration (72 h). At 72 hpi, cells were fixed and stained for JCPyV VP1 expression for ICW and FFU as previously described for each assay type. For each experimental assay, triplicate samples were analyzed and the percent infection or percent response for each treatment was normalized to the DMSO control, which was set to 100%. Error bars represent the standard deviation from the mean. ^∗^P < 0.05. Data are representative of three independent experiments.

To characterize viral infectivity in the presence of the aforementioned MAPK inhibitors, SVG-A cells were pretreated with DMSO (vehicle control), Bay43-9006, PD98059, or U0126 and subsequently infected with JCPyV (Figure 2). A cell viability assay (MTS) was used to confirm that chemical inhibitors were not toxic (data not shown) and total cell numbers are accounted for through CellTag (ICW) and DAPI staining (FFU). Cells were fixed and stained for VP1 using both FFU and ICW assays performed in parallel. SVG-A cells treated with the inhibitor Bay43-9006 demonstrated an ∼60% decrease in infection in the case of both the FFU and ICW analyses, in comparison to controls (Figure 2A). The MEK inhibitor PD98059 decreased JCPyV infectivity by ∼80% as measured by FFU and ICW (Figure 2B). U0126 reduced JCPyV infectivity by ∼60% in both the FFU analysis and the ICW quantitation of JCPyV VP1 expression (Figure 2C). In addition, the retrograde trafficking inhibitor Retro2, which has been previously demonstrated to inhibit JCPyV infection (Nelson et al., 2013), was tested as a JCPyV inhibitor via ICW. Treatment of SVG-A cells with Retro2 significantly reduced JCPyV infection by ∼90% as measured by FFU and ∼80% by ICW (Figure 2D), suggesting that an inhibitor of another component of the viral infectious cycle, viral trafficking, can be assessed by ICW. These data demonstrate the versatility of the ICW analysis in the characterization of viral infection during chemical inhibition.

### Assessment of JCPyV Infectivity Following Protein Silencing

**FIGURE 3:**
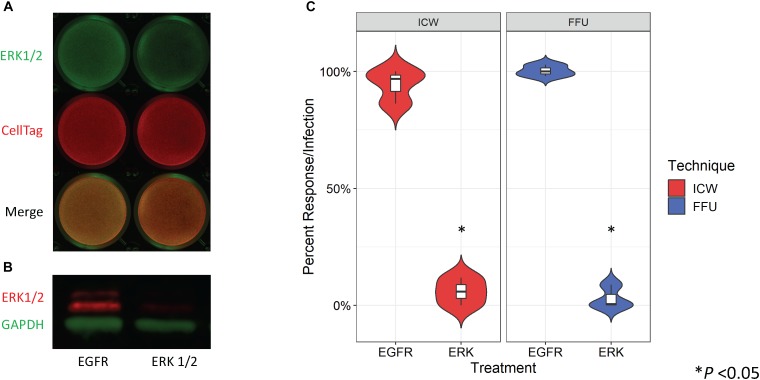
Quantitation of ERK siRNA inhibition of JCPyV infection by ICW. SVG-A cells were transfected with either an irrelevant EGFR control or ERK1/2 siRNA and incubated at 37°C for 72 h. **(A)** ICW analysis of the siRNA-transfected cells was used to confirm ERK1/2 knockdown using an antibody targeting ERK1/2 (green) and CellTag (red) to normalize to the total number of cells present. **(B)** Western blot analysis of cell lysates from siRNA-transfected cells were also analyzed to confirm ERK1/2 knockdown, using antibodies specific for ERK1/2 (red) and GAPDH (green) (loading control). **(C)** At 72 hpt, siRNA-transfected cells were infected with JCPyV (MOI = 0.5 FFU/cell) at 37°C for 1 h and then fed with cMEM for 1 hpi. At 72 hpi, FFU and ICW plates were fixed and stained as previously described for each assay type for JCPyV VP1 expression. Distribution trends between triplicate samples are represented by violin plot for both ICW and FFU. ^∗^P < 0.05. Data are representative of three independent experiments.

Treatment of SVG-A cells with an ERK1/2-specific siRNA has been previously shown to significantly decrease JCPyV infection as measured by FFU quantitation (DuShane et al., 2018). To determine whether siRNA-induced protein knockdown can be detected by the ICW, SVG-A cells were transfected with either an irrelevant EGFR- or an ERK1/2-specific siRNA. In parallel, siRNA transfected SVG-A cells under the same conditions were also processed by western blot to confirm that the ICW provides an accurate measure of protein expression. ERK protein expression was reduced in samples transfected with the ERK1/2 siRNA in comparison to the EGFR control for both the ICW and western blot assays (Figure 3A,B), suggesting that the ICW is a viable method for protein expression analysis during siRNA transfection.

To determine if the ICW can be used to quantitate JCPyV infection during siRNA conditions, SVG-A cells were transfected with the aforementioned siRNAs and cells were subsequently infected with JCPyV. Quantification of VP1 by FFU and ICW showed significant decreases of ∼90% for both percent infection and percent response (Figure 3C), suggesting that the ICW can be utilized to quantitate JCPyV infection during siRNA transfection with the same degree of accuracy as traditional FFU assays.

### JCPyV Infection Impacts on Host-Cell Protein Expression

**FIGURE 4:**
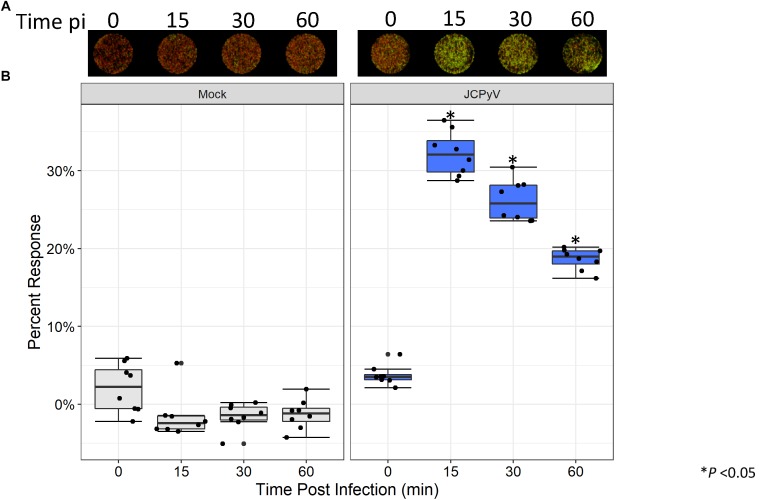
JCPyV-induced ERK activation as measured by ICW. **(A)** SVG-A cells were mock infected (cMEM without virus) or infected with JCPyV (MOI = 0.5 FFU/cell) for the specified duration and were then fixed and subsequently stained using antibodies targeting pERK1/2 and CellTag (to normalize to the total number of cells). **(B)** The percentage of pERK at each timepoint was quantitated by ICW signal intensity values per [(pERK/Cell Tag) × 100%]. Data from nine samples are represented by box and whisker plot depicting the median and first and third quartiles. Dots represent individual points comprising box and whisker plots. Whiskers represent values 1.5 times the distance between the first and third quartiles (inter-quartile range). ^∗^P < 0.05 comparing JCPyV- to mock-infected samples at specified time points. Data are representative of three independent experiments.

Previous research has demonstrated that early events during JCPyV infection induce alterations to the MAPK pathway resulting in phosphorylation of ERK (Querbes et al., 2004; DuShane et al., 2018). Within 15 min of viral challenge, pERK is highly upregulated in SVG-A cells (Querbes et al., 2004; DuShane et al., 2018). Traditionally, quantitation of protein phosphorylation has been measured through western blotting techniques, which can be laborious and time consuming. However, the ICW has been implemented as an effective tool for the quantitation of both protein expression and signal transduction (Hannoush, 2008; Aguilar et al., 2010; Schnaiter et al., 2014; Cuartas-López et al., 2018), but has had limited use in conjunction with the study of viral infectivity (Cuartas-López et al., 2018). We employed the ICW technique to determine if alterations to pERK levels during JCPyV infection could be quantified using the ICW methodology, SVG-A cells were either mock-infected or infected with JCPyV, and levels of pERK were assessed by ICW over a time course of infection. At 15 min post viral challenge, activation of ERK peaked, followed by a steady decline through 1 hpi (Figure 4). These findings are in line with previously published work demonstrating that levels of pERK are highly upregulated in JCPyV-infected samples in comparison to mock-infected SVG-A cells at early timepoints during infection (Querbes et al., 2004; DuShane et al., 2018). These results demonstrate that the ICW can not only assess viral infectivity, but can also quantify host-cell protein response to viral infection.

### ICW Analysis of Infectivity by Other Polyomaviruses

**FIGURE 5:**
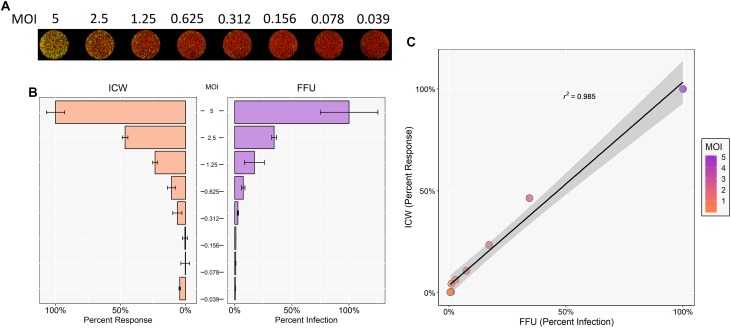
ICW characterization of SV40 polyomavirus. SVG-A cells were infected with SV40 at indicated MOIs for 72 h. Cells were then fixed and stained for VP1 expression. SV40 VP1 expression was analyzed by ICW and FFU quantitation as in Figure 1. **(A)** ICW plates were stained with CellTag 700 (red) and a LI-COR-800 secondary antibody to target VP1 (green). **(B)** For each experimental assay, a minimum of triplicate samples were analyzed and the percent infection or percent response for each treatment was normalized to the DMSO control, which was set to 100%. For each experimental assay, the percent infection or percent response of a given MOI was normalized to the highest MOI, which was set to 100%. Error bars are representative of the standard deviation from the mean. **(C)** ICW and FFU data were compared across identical conditions by linear regression and an experimental correlation was determined using a Pearson correlation coefficient. The gray shaded region represents the confidence interval (standard error of the linear regression). Error bars represent the standard deviation from the mean. Data are representative of three independent experiments.

SV40 is the most phylogenetically related polyomavirus to JCPyV and has a very similar replication cycle (Perez-Losada et al., 2006). Thus, it was hypothesized that ICW can be utilized to quantify and characterize SV40 infectivity as well. To determine whether the ICW technique could be applied to SV40, SVG-A cells were infected with varying MOIs of SV40. Cells were then incubated for 72 h and stained for SV40 VP1 expression and analyzed by FFU and ICW (Figure 5A,B). Resultant data from both FFU and ICW demonstrated an increase in VP1 expression that correlated with increasing MOI (Figure 5C). Moreover, infectivity quantified by FFU and ICW proved to be positively correlative as per a Pearson correlation coefficient of 0.985, suggesting that the ICW is a viable and accurate measure of SV40 infectivity (Figure 5C).

## Discussion

The study of viral infections in vitro has provided innumerable advances to the field of virology. However, the lack of rapid and efficient screening tools has hindered research progress for some viruses, like JCPyV (Houff et al., 1983; Zu Rhein, 1983; Assetta and Atwood, 2017). To overcome this challenge, the development of high-throughput analyses is needed to help aid in the production of large data sets and generation of multiple lines of inquiry. Current methodologies for analyzing JCPyV infectivity predominantly rely on manual quantitation of infection by indirect immunodetection of viral proteins by epifluorescence microscopy. While these methodologies have provided crucial information into the characterization of JCPyV infectivity, the low-throughput nature of microscopy-based techniques has prevented the employment of large-scale screens and also hindered productivity within the field. Adapting the ICW procedure to effectively measure JCPyV infectivity has enabled faster analysis by improving efficiency by ∼9 person hours per 96-well plate in comparison to traditional FFU manual assessments. Moreover, this semi-automated assay allows for enhanced user objectivity. Utilization of ICW analysis to detect JCPyV infection and viral impacts on host-cell proteins provides a new technological platform for large-scale screens to measure the viral response to treatments such as inhibitors or siRNAs with enhanced efficiency and reduced use of resources.

The results described herein demonstrate that the ICW provides a rapid, robust, and accurate measure of infectivity and can also assess viral impacts on host-cell protein expression. Previous work has shown that the ICW can be utilized for quantifying viral infectivity of multiple types of viruses, including influenza, herpes simplex virus, reovirus, and rotavirus (Wan et al., 2010; Iskarpatyoti et al., 2012; Fabiani et al., 2017). To confirm that the ICW can be used to measure JCPyV infectivity with accuracy, SVG-A cells were subjected to JCPyV infection at varying MOIs for both FFU and ICW quantitation (Figure 1). Both assays demonstrated increasing detection of VP1 with increasing MOIs, demonstrating a positive Pearson correlation coefficient (Figure 1). The positive correlation between the traditional microscopic quantitation of JCPyV and the ICW approach indicates that the ICW is a reliable and accurate technique to characterize JCPyV infectivity. However, a limitation of the ICW includes the quantifiable limit of viral protein expression, particularly when administering low viral MOIs for infectivity assessment. For most JCPyV-associated experiments, using a MOI of 0.1 FFU/cell and greater produced more representative and reproducible results, suggesting that this may be the lower limit of detection for this assay when assessing JCPyV infectivity by VP1 expression. Together these findings confirm that the ICW can be utilized to quantitate JCPyV infection in SVG-A cells (Figure 1) and as such, JCPyV infectivity can be accurately quantitated using this novel methodology.

Due to viral dependency on the host cell for infectivity, virus–host cell interactions are a key area of focus in virology research. To explore whether viral inhibition can be measured by ICW during JCPyV infection, SVG-A cells were exposed to chemical inhibitors targeting endocytic pathways and the MAPK cascade. Under all treatments, as measured by both FFU and ICW, JCPyV infectivity was significantly reduced in comparison to controls (Figure 2) suggesting that ICW is a viable option for screening potential anti-viral compounds. Moreover, JCPyV infectivity can be scored in response to siRNA knockdown of proteins known to significantly decrease infection (DuShane et al., 2018), as VP1 expression was significantly decreased in cells lacking ERK1/2 as measured by both FFU and ICW (Figure 3). Importantly, these findings demonstrate that the ICW is capable of detecting changes in JCPyV infectivity following siRNA inhibition supporting its use in conjunction with high-throughput drug and siRNA screening libraries.

JC polyomavirus has been previously shown to upregulate the critical MAPK signaling molecule ERK early in the infectious process to facilitate viral infection (Querbes et al., 2004; DuShane et al., 2018). Through the ICW, alterations to normal expression or activation of ERK and other proteins can be quantitated in this plate-based system faster and more reproducibly to alternative traditional western blotting methods (Boveia et al., 2009; Boveia and Schutz-Geschwender, 2015). While this technique has been previously used to detect phosphoproteins, it has had limited adaptations with virology-based studies. We have adapted this sensitive and quantitative measure of protein phosphorylation to assess viral impact on host-protein expression. Quantitation of pERK by ICW demonstrated that JCPyV-induced activation of ERK was robust at early timepoints during infection (Figure 4), in line with previously published results (DuShane et al., 2018), suggesting that the ICW can accurately detect host-cell protein changes during viral infection. The ICW provides an advantage over traditional western blot techniques as cells can be fixed under more controlled intervals and processed directly within the sample plate. These findings highlight the capacity of this assay to analyze changes in host-cell protein response due to viral infection, suggesting it can be successfully applied to large-scale studies of viral-induced proteomic alterations.

In addition, the ICW can also be adapted to measure infectivity of other polyomaviruses like SV40 PyV (Perez-Losada et al., 2006). SVG-A cells infected with SV40 quantified by both FFU and ICW demonstrated a corresponding increase in both percent infection and percent response with increasing MOIs, which proved to be positively correlated (Figure 5). These findings indicate that SV40 infection may also be characterized by the ICW assay and suggest that this assay could be adapted for the detection of other polyomaviruses such as BK polyomavirus. Thus, this assay can be further validated and developed for other viruses and cell types. Together these results demonstrate the dynamic range of experimental designs that can be investigated using the ICW. With a rapid and automated approach, a significant amount of experimental information can be generated under high-throughput conditions and analyzed quickly and efficiently with this novel technology.

The ICW assay was initially designed to study protein expression in lieu of the traditional western blot system (Boveia and Schutz-Geschwender, 2015). This system captures information regarding proteins of interest in a context that is directly pertinent to the cell, in a matter of hours rather than days. Since its implementation, it has been adapted to study viral protein production during the infectious process and characterize viral infection under different experimental conditions. This high-throughput application provides the means by which researchers can generate large data sets that can help drive productive research forward in a quick, unbiased manner, which can now be applied to the study of JCPyV. The addition of this new technology for characterizing JCPyV provides an advance to the field as the current methods for detection are limited to microscopy-based FFU assays, as JCPyV can neither be measured via plaque assay, nor studied in small animal model systems (Zu Rhein and Varakis, 1979; Calgua et al., 2011). Thus, the findings described herein demonstrate that the ICW provides a unique high-throughput platform for quantifying viral infectivity that can be utilized to increase the rate of discovery and drive the study of *Polyomaviridae* forward.

## Author Contributions

JD and MM conceived the study. JD designed the experiments. JD and MC performed the experiments. JD, MC, MW, and MM evaluated the data. JD and MW performed data analysis and prepared figures. JD and MM wrote the manuscript. All authors contributed to manuscript editing and approved the final submitted manuscript.

## Conflict of Interest Statement

The authors declare that the research was conducted in the absence of any commercial or financial relationships that could be construed as a potential conflict of interest.
